# Diabetes‐Associated Cognitive Impairment: Pathophysiology and Emerging Therapies

**DOI:** 10.1002/acn3.70487

**Published:** 2026-10-11

**Authors:** Osman Corbali, Priyanka Menon, Halil I. Corbali, Ram Jagannathan, Allan I. Levey

**Affiliations:** ^1^ Department of Neurology Emory University School of Medicine Atlanta Georgia USA; ^2^ Medical College of Georgia Augusta University Augusta Georgia USA; ^3^ Massachusetts General Hospital Harvard Medical School Boston Massachusetts USA; ^4^ Emory Global Diabetes Research Center Woodruff Health Sciences Center and Emory University Atlanta Georgia USA; ^5^ Hubert Department of Global Health Emory University Atlanta Georgia USA

**Keywords:** Alzheimer’s disease, brain insulin resistance, neuroinflammation, neurovascular dysfunction, vascular dementia

## Abstract

Diabetes‐associated cognitive impairment (DACI) is a common yet underrecognized condition, manifesting along a spectrum from mild cognitive impairment to dementia. DACI encompasses patients who develop vascular dementia (VaD) or Alzheimer's disease (AD) with diabetes as a risk factor; however, in certain patients, particularly those lacking clear VaD or AD features, diabetes may act as a primary driver of neurodegeneration. Mechanistically, DACI can be conceptualized as a neurovascular‐metabolic disorder characterized by disruption of the neurovascular unit, impaired cerebral glucose metabolism, brain insulin resistance, and chronic neuroinflammation, contributing to small vessel disease, regional brain atrophy, and accelerated brain aging. In this narrative review, we synthesize current evidence on the clinical and imaging features of DACI, outline key mechanistic pathways linking diabetes to neurodegeneration, and discuss emerging therapeutic strategies, including GLP‐1 receptor agonists and SGLT2 inhibitors, with potential neuroprotective effects. Greater recognition of DACI as a neurovascular‐metabolic entity may facilitate earlier identification, inform biomarker development, and guide future trials aimed at preserving cognitive function in individuals with diabetes.

## 1 Introduction

Diabetes‐associated cognitive impairment (DACI) is a spectrum of cognitive dysfunction ranging from mild cognitive impairment to dementia. DACI encompasses patients who develop vascular dementia (VaD) or Alzheimer's disease (AD) with diabetes as a risk factor; however, in certain patients, particularly those lacking clear VaD or AD features, diabetes may act as a primary driver of cognitive impairment rather than a mere contributor (Figure 1A) [1]. Cognitive disorders are common among individuals with diabetes, with a recent meta‐analysis estimating a pooled prevalence of approximately 40%, although estimates vary by population and cognitive assessment method [2]. With an estimated 828 million adults living with diabetes globally [3], the potential burden of DACI is substantial and is observed across both middle‐aged and older adults [4, 5, 6, 7].

**FIGURE 1:**
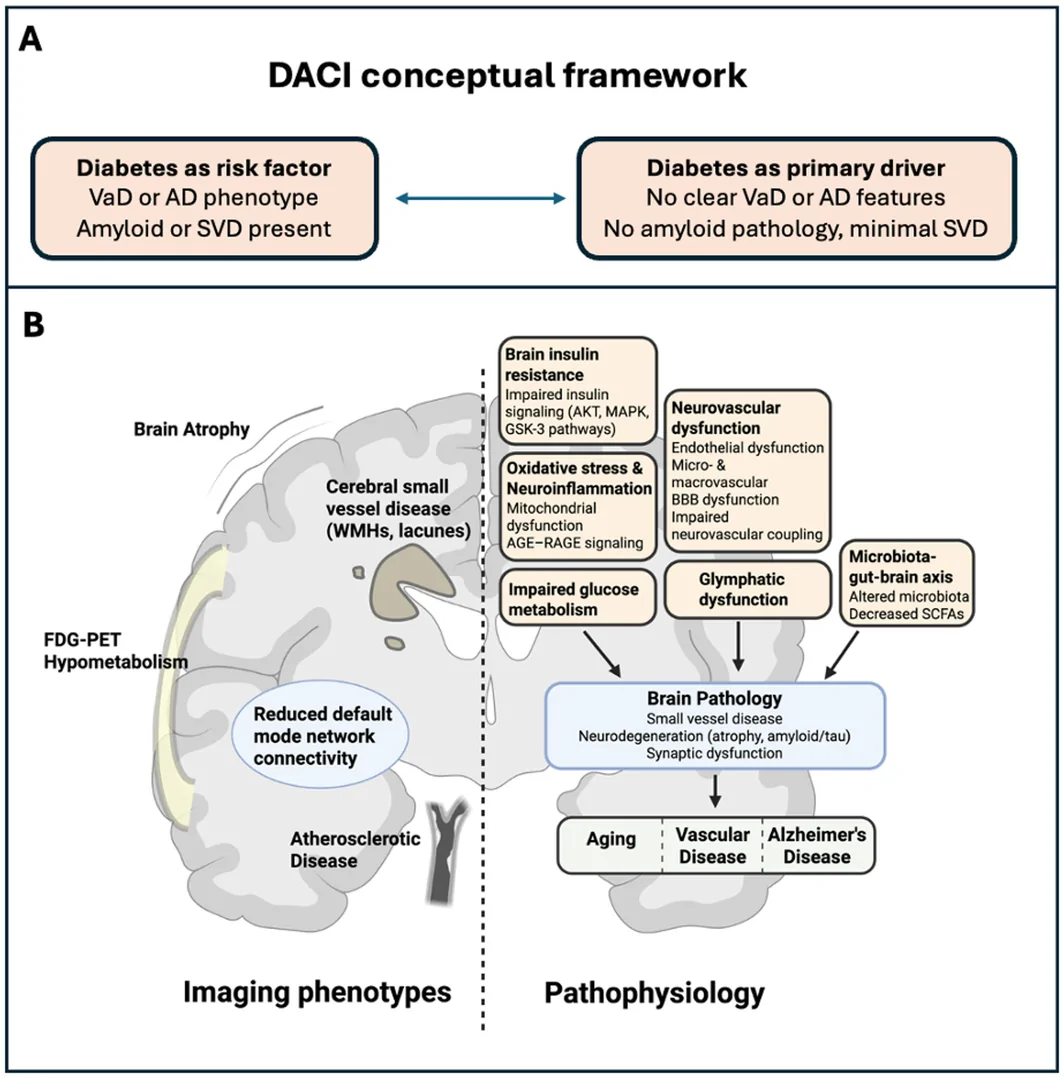
DACI conceptual framework, imaging features, and pathophysiology. The top panel (A) illustrates the proposed DACI spectrum. The bottom panel (B) illustrates characteristic imaging features, including cerebral small vessel disease, brain atrophy, FDG‐PET hypometabolism, atherosclerotic disease (intracranial or extracranial), and reduced default mode network connectivity. The right panel summarizes proposed pathophysiological mechanisms, including brain insulin resistance, impaired glucose metabolism, oxidative stress, neurovascular dysfunction, glymphatic dysfunction, and gut–brain axis alterations, converging on neurodegeneration and cognitive decline. AD, Alzheimer's disease; AGE, advanced glycation end products; BBB, blood–brain barrier; FDG‐PET, Fluorodeoxyglucose positron emission tomography; RAGE, receptor for advanced glycation end products; SCFAs, short‐chain fatty acids; SVD, small vessel disease; VaD, Vascular dementia; WMH, white matter hyperintensity.

The risk of dementia with diabetes increases with greater diabetes severity, longer disease duration, history of hypoglycemia, or the presence of comorbid conditions [8, 9, 10, 11]. Each 1% increase in HbA1c is associated with an 8% higher risk of developing dementia [11]. Although optimal diabetes management improves cardiometabolic outcomes, intensive glucose‐lowering strategies (targeting HbA1c < 6% or < 6.5%) have not conferred significant cognitive benefit compared to standard approaches [12, 13, 14], suggesting that hyperglycemia alone does not fully explain DACI.

DACI arises from the interplay of multiple mechanisms, including neurovascular dysfunction, brain insulin resistance, and chronic neuroinflammation, resulting in brain changes that partially overlap with those observed in Alzheimer's disease (AD) [5, 15, 16]. Understanding the pathophysiology of DACI, and its shared mechanisms with AD, is therefore critical, as therapies targeting convergent pathways may offer neuroprotective potential across both conditions. In this narrative review, we summarize (a) the clinical and radiological features of DACI, (b) key pathophysiological mechanisms underlying DACI, and (c) emerging therapeutic strategies informed by these mechanistic insights. This review primarily focuses on Type 2 diabetes (T2D), which accounts for the majority of diabetes cases worldwide (about 96%) [17].

## 2 Diabetes‐Associated Cognitive Impairment: Clinical and Radiological Features

### 2.1 Clinical Features

DACI commonly manifests close to a vascular cognitive impairment phenotype, with executive function and processing speed predominantly affected, though memory impairment is also frequently observed [2, 18, 19, 20, 21, 22]. Diabetes increases the risk of both vascular dementia [RR = 1.58; 95% CI: 1.48–1.69] and Alzheimer's disease [RR = 1.30; 95% CI: 1.11–1.53] [15, 23, 24]. Even prediabetes has been linked to a higher risk of cognitive impairment and dementia, including both vascular and Alzheimer's subtypes [24]. In longitudinal studies of patients with MCI, diabetes consistently increases conversion to vascular dementia, while its association with conversion to Alzheimer's disease remains mixed across studies [25, 26, 27]. Importantly, cognitive deficits and increased brain atrophy can be detected in young and middle‐aged adults with diabetes, even before a dementia diagnosis [4].

Diabetes is also associated with other neurodegenerative diseases of aging. These include an increased risk of Parkinson's disease and faster motor progression in affected individuals [28, 29]. Evidence regarding dementia with Lewy bodies remains mixed [30, 31, 32, 33], and data on frontotemporal dementia are limited, with one study suggesting a possible increased risk [34].

### 2.2 Radiological Features

Among imaging findings, cerebral atrophy, cerebral small vessel disease, and glucose hypometabolism are the most consistently reported in diabetes (Figure 1B). Cross‐sectional and longitudinal studies have reported reductions in total brain volume [4, 35]. Regional brain atrophy, including in the hippocampus, frontal, temporal, and occipital lobes, has been variably reported [4, 35, 36, 37].

Diabetes increases the risk of small‐vessel disease, manifesting as silent brain infarcts and white‐matter hyperintensities [38, 39]. The periventricular distribution pattern, commonly observed in diabetes‐related small vessel disease, is generally considered more consistent with a vascular origin, though the etiology of white matter hyperintensities remains debated, with both vasculogenic and Wallerian degeneration mechanisms proposed [40, 41]. Importantly, the severity of small vessel disease in diabetes is an important predictor of future cognitive decline [42, 43]. In addition, white‐matter microstructure is altered; diffusion tensor imaging studies demonstrate lower fractional anisotropy and higher mean diffusivity in diabetic patients with mild cognitive impairment compared with diabetic patients with normal cognition [44].

Functional connectivity is likewise affected. Resting‐state fMRI studies demonstrate reduced connectivity within the default mode network, a pattern also observed in aging and dementia [45, 46]. In contrast, task‐based fMRI studies have yielded more variable results [46].

Alterations in cerebral blood flow (CBF) have also been reported, with decreases in global and regional CBF observed in individuals with diabetes; however, these findings are less consistent after accounting for concurrent brain atrophy [47, 48, 49]. Neurovascular coupling (NVC), the relationship between neuronal activity and CBF, is disrupted in diabetes and discussed in greater detail in the next section [50], along with metabolic and molecular imaging findings including FDG‐PET hypometabolism and amyloid and tau PET abnormalities.

## 3 Pathophysiology of Diabetes‐Associated Cognitive Impairment

Cognitive decline in diabetes is driven by neurovascular dysfunction, brain insulin resistance, and hyperglycemia. Together these processes lead to mitochondrial dysfunction, oxidative stress, and neuroinflammation, creating a pathological profile that closely resembles Alzheimer's disease (Figure 1B) [15, 16, 51, 52]. Vascular injury, including atherosclerosis, cerebral small vessel disease, and disruption of the blood–brain barrier (BBB), adds to the burden [39, 51].

### 3.1 Neurovascular Dysfunction

Vascular injury in diabetes involves endothelial dysfunction, microvascular pathology, macrovascular disease, BBB impairment, and abnormalities in cerebral blood flow [38, 53]. These changes are worsened by common diabetes comorbidities such as hypertension, obesity, and hyperlipidemia.

#### 3.1.1 Endothelial Dysfunction

Endothelial dysfunction is an early sign of vascular compromise in diabetes, and it has been demonstrated by reduced flow‐mediated dilation in individuals with diabetes [54, 55]. Endothelial cells rely on insulin‐independent GLUT1 and GLUT3‐mediated glucose transport, which can expose cells to excess intracellular glucose during hyperglycemia [56]. Hyperglycemia subsequently induces oxidative stress through mitochondrial dysfunction, activates chronic inflammatory pathways (including nuclear factor‐κB [NF‐κB]), reduces nitric oxide bioavailability, and promotes endothelial‐to‐mesenchymal transition (EndMT) via TGF‐β signaling as well as endothelial cell death [55]. Together, these processes contribute to endothelial injury.

#### 3.1.2 Atherosclerosis

Persistent endothelial damage accelerates macrovascular and microvascular complications. Advanced glycation end products (AGEs) and inflammatory mediators upregulate adhesion molecules on the endothelium, promoting monocyte and macrophage infiltration into the subendothelial space [55, 57]. Hyperglycemia can also glycate low‐density lipoprotein (LDL) particles, increasing their atherogenicity [58]. Additional mechanisms promoting atherosclerosis include EndMT and phagocytosis of glycated erythrocytes, which destabilize plaques [59, 60].

Diabetes promotes both extracranial and intracranial atherosclerotic disease [61, 62], and accordingly the risk of ischemic stroke is more than twofold higher in individuals with diabetes, even after adjusting for common comorbidities including smoking, BMI, systolic blood pressure, and dyslipidemia [63].

#### 3.1.3 Neurovascular Coupling

NVC refers to the dynamic adjustment of CBF in response to neuronal activity and metabolic demand. This process is mediated by the neurovascular unit, which comprises neurons, astrocytes, and components of the cerebral vasculature, including endothelial cells, smooth muscle cells, pericytes, and the BBB [64]. Growing evidence from cross‐sectional human studies indicates that NVC is altered in diabetes [65, 66, 67, 68, 69], reflecting disrupted coordination between neuronal activity and cerebral perfusion, including within DMN hubs [66, 67]. In addition, diabetes may accelerate NVC abnormalities over time [70].

#### 3.1.4 Orthostatic Hypotension

Orthostatic hypotension (OH) is also a risk factor for cognitive impairment [71]. It is commonly a manifestation of autonomic neuropathy in diabetes and is nearly twice as common (approximately 25%) in individuals with diabetes compared to the general population [72, 73]. Patients with diabetes and OH have been shown to exhibit lower cognitive performance across multiple domains in the standing position compared to diabetic patients without OH [74]. Furthermore, the presence of OH in diabetes has been associated with an increased risk of dementia, Alzheimer's disease, and vascular dementia [75].

#### 3.1.5 Blood Brain Barrier Impairment

Diabetes is also associated with BBB disruption [38]. This is evidenced by basement membrane thickening, endothelial dysfunction, and pericyte degeneration [76]. BBB impairment is also an early feature of Alzheimer's disease and may precede both brain atrophy and cognitive symptoms [77]. Preclinical data indicate that BBB dysfunction promotes neuroinflammation and glial activation [78]. These processes appear relevant in diabetes as well, yet the specific role of BBB disruption in mediating cognitive decline remains uncertain.

### 3.2 Brain Insulin Resistance

Brain insulin resistance refers to reduced responsiveness of neurons and glia to insulin, resulting in impaired synaptic plasticity, deranged energy metabolism, and dysregulated neuroimmune signaling [79]. It is a shared feature of AD and T2D and is influenced by aging and peripheral insulin sensitivity [79, 80, 81].

Insulin crosses the BBB through a saturable transporter system, the efficiency of which is modulated by obesity, aging, diabetes, and neurodegeneration [79, 82]. In animal models, insulin transport is reduced in obesity, aging, and AD, but paradoxically increased in diabetes [82, 83]. In humans, the CSF‐to‐serum insulin ratio positively correlated with peripheral insulin sensitivity, suggesting reduced insulin transport across the BBB with greater insulin resistance [84]. Although insulin is also locally expressed in the brain, endogenous production is minimal and of uncertain physiological significance [79, 85].

Within the CNS, insulin acts predominantly via IRS‐AKT and IRS‐MAPK signaling cascades in neurons and astrocytes [79]. Insulin signaling supports neurite outgrowth [86], inhibits apoptosis [79], enhances mitochondrial function [87, 88], and modulates synaptic plasticity [79, 86]. Postmortem studies in AD and T2D brains reveal abnormalities in insulin‐related pathways, including IRS1–AKT–mTOR and GSK3, consistent with impaired insulin signaling [89, 90, 91]. In AD, postmortem measures of central insulin resistance correlate positively with amyloid‐β and tau pathology and inversely with cognitive performance [92, 93].

In vivo, brain insulin resistance is assessed using intranasal insulin administration combined with neuroimaging or neuron‐derived extracellular vesicles, though further research is needed to establish validated biomarkers for brain insulin resistance in diabetes and AD [94].

### 3.3 Impaired Cerebral Glucose Metabolism

Glucose metabolism is disrupted in diabetes, VaD, and AD, as demonstrated by reduced global and regional glucose uptake on PET imaging [95, 96, 97, 98, 99, 100, 101, 102]. FDG‐PET can successfully differentiate AD from VaD based on distinct regional patterns: AD typically involves the temporoparietal lobes, posterior cingulate cortex, and mesial temporal lobe, whereas VaD shows a different pattern that affects the frontal lobes and deep nuclei [101, 102]. Interestingly, diabetes is also associated with cerebral glucose hypometabolism on FDG‐PET; however, the distribution varies considerably across studies [100]. In a twin‐discordant study, diabetes was associated with global glucose hypometabolism [99]; an MCI cohort from the ADNI database showed decreased metabolism in the frontal lobe, sensorimotor cortex, and striatum [103]; and another study reported hypometabolism overlapping with AD signature regions identified by amyloid PET [104]. This heterogeneity is intriguing, and whether FDG‐PET patterns in DACI can be systematically clustered into AD‐like, vascular, or diffuse metabolic subtypes remains an important unanswered question.

Under normal physiological conditions, glucose crosses the BBB via GLUT1 transporters in endothelial cells, subsequently diffusing into the parenchyma or being taken up by astrocytes through the same transporter [97, 105, 106, 107]. Neuronal glucose uptake occurs primarily through GLUT3, an insulin‐independent transporter, whereas GLUT4 facilitates additional uptake during heightened metabolic demand [79, 107, 108, 109, 110]. Endothelial GLUT1 expression is essential for BBB integrity and cerebral glucose delivery, and its reduction impairs cerebral perfusion and diminishes amyloid‐β clearance [106].

In AD, reduced cerebral glucose transport, expression of GLUT1 and GLUT3 as well as glucose metabolism (FDG‐PET) have been shown [97, 98, 111]. Furthermore, lower levels of GLUT1 and GLUT3 correlated with hyperphosphorylated tau [111]. Another study, using proteomic and metabolomic analysis, showed that advancing AD pathology (as measured by Braak or CERAD staging) is associated with elevated brain tissue glucose, decreased glycolytic flux, and reduced GLUT3 expression [112]. At the same time, preclinical work demonstrates that soluble Aβ peptides inhibit glucose transport at the BBB and reduce GLUT1 expression in vivo, suggesting glucose transport may have a bidirectional relationship with AD pathology [113].

In diabetes, disturbances in brain glucose transport require further investigation. Human MR‐spectroscopy studies demonstrate a blunted rise in brain glucose levels during hyperglycemia in individuals with diabetes compared with those with normal glucose regulation, suggesting the possibility of impaired glucose transport [114]. However, whether glucose transporter expression is altered in humans remains unclear. Preclinical studies indicate that acute hyperglycemia downregulates endothelial GLUT1 [115, 116], whereas chronic diabetes does not [116, 117], implying that protective adaptations in GLUT1 expression may be lost or altered in long‐standing diabetes [116].

### 3.4 Amyloid and Tau Pathology

In human brain pathology, most studies have found no association between diabetes and AD‐related neuropathological changes, including neuritic plaques (Aβ) or neurofibrillary tangles (intracellular tau aggregates) [118, 119, 120, 121, 122, 123, 124, 125, 126]. Even in patients with dementia, diabetes was not associated with increased neuritic plaques or neurofibrillary tangles on pathology [127]. Cross‐sectional PET studies have likewise not identified significant differences in amyloid deposition between individuals with diabetes and non‐diabetic controls [104, 128, 129]. Exceptions include the Hisayama study, in which insulin resistance and hyperinsulinemia were associated with AD pathology at autopsy [130]; the Rotterdam study, in which diabetes assessed 7 years prior to amyloid‐PET was associated with amyloid positivity (whereas diabetes assessed 12 years before was not) [131]; and a few cross‐sectional studies that reported associations between amyloid deposition on PET and prediabetes or insulin resistance [129, 131, 132]. Another amyloidogenic peptide, amylin (islet amyloid polypeptide), which is synthesized in pancreatic β‐cells and elevated in insulin‐resistant states, has been detected in the brains of individuals with diabetes [133], however, human data remain limited, and the extent to which amylin directly contributes to DACI remains uncertain.

Diabetes may instead preferentially modify tau pathology. Mechanistically, insulin resistance may influence tau pathology; due to impaired activation of the PI3K/Akt pathway, GSK‐3β remains active to phosphorylate tau [134]. CSF‐based studies show mixed results; one ADNI analysis reported elevated CSF tau (phosphorylated and total) in T2D while another found no significant difference [128, 135]. An exploratory multicohort analysis suggested enrichment of amyloid‐negative tau‐positive CSF profiles in individuals with diabetes compared to those without, in both cognitively normal and MCI participants [136]. In a PET imaging study of individuals with diabetes‐related dementia, 11 of 21 participants demonstrated a tau‐positive but amyloid‐negative pattern, while 4 of 21 were negative for both amyloid and tau deposition [137]. In another ADNI analysis, baseline regional tau PET did not differ by diabetes status; though diabetes was longitudinally associated with greater hippocampal tau deposition relative to amyloid burden [138]. Similarly, in a subgroup analysis of the Framingham Heart Study (*n* = 288; 2.8% with diabetes), higher fasting plasma glucose levels predicted increased temporal tau deposition on PET 14 years later, while no significant association with amyloid‐β deposition was observed [139].

The discrepancy between neuropathological findings and neuroimaging or CSF‐based tau measures raises an important question: whether diabetes promotes an earlier, pre‐tangle form of tau pathology, reflected by elevated p‐tau in CSF and tau PET signal in a subset of patients, rather than the mature neurofibrillary tangle burden characteristic of advanced AD. This distinction merits dedicated longitudinal investigation.

### 3.5 Oxidative Stress and Neuroinflammation

The brain is highly susceptible to oxidative injury due to its disproportionately high energy demands and high lipid content [140, 141]. Diabetes contributes to this oxidative injury through excessive reactive oxygen species (ROS) generation, mitochondrial dysfunction, and neuroinflammation [140, 142, 143, 144, 145, 146, 147].

#### 3.5.1 AGE‐RAGE Signaling

Diabetes is associated with increased circulating advanced glycation end products (AGEs) levels in serum [148, 149, 150], and preliminary data have shown a strong correlation between serum and brain AGE levels in a small post‐mortem study [148]. Interactions between AGEs and the receptor for advanced glycation end products (RAGE) activate multiple signaling pathways, including NF‐κB, MAPK, PI3K/AKT, and GSK‐3β [151, 152, 153, 154]. This interaction can lead to microglial activation, contributing to ROS generation, proinflammatory cytokine release, and increased amyloid‐β pathology and tau hyperphosphorylation in experimental models [155, 156, 157, 158]. In the periphery, RAGE signaling may further disrupt insulin pathways, linking AGE burden to insulin resistance [159, 160].

#### 3.5.2 Microglial Activation

In preclinical diabetes models, microglia polarize toward a pro‐inflammatory M1 phenotype, releasing cytokines such as IL‐1β, IL‐17A, and TNF‐α, trigger apoptotic cascades, and may promote amyloid deposition [161, 162, 163, 164, 165, 166]. In humans, the microglial phenotype in diabetes remains to be characterized. Notably, higher insulin resistance (HOMA‐IR) and elevated BMI have been associated with increased cortical TSPO‐PET signal, indicative of microglial activation, in cognitively unimpaired older adults [167, 168]. TSPO‐PET also demonstrates microglial activation across multiple neurodegenerative diseases and shows strong associations with tau pathology in AD [169, 170].

#### 3.5.3 Ferroptosis

Ferroptosis, a non‐apoptotic, iron‐dependent form of cell death, has been implicated in both diabetes and AD [171]. Patients with diabetes or AD exhibit higher iron levels in various brain regions, as measured by MRI‐based methods [172, 173, 174], and iron accumulation appears to correlate with amyloid pathology in AD [175].

### 3.6 Synaptic Dysfunction

Insulin signaling plays an important role in synaptic plasticity and modulates neurotransmission by regulating ion channels and receptor trafficking, including GABA, NMDA, and AMPA receptors, thereby influencing long‐term potentiation and depression [79, 176]. In diabetic murine models, alterations in brain metabolite levels, including decreased glutamate and glutamine levels, have also been reported [177]. Insulin also regulates dopaminergic transmission: in experimental models, insulin increases both dopamine reuptake and release from the neurons (e.g., in the ventral striatum), and insulin resistance can blunt this dopaminergic pathway [178].

### 3.7 Brain Aging

In diabetes, neuropsychological and radiological features of accelerated aging have been described, including reduced processing speed, executive function, and regional brain atrophy [179]. Molecular biomarkers of brain aging remain an area of active exploration; epigenetic aging markers based on DNA methylation, such as the Cortical clock and PCBrainAge, have not been investigated in diabetes specifically, to the best of our knowledge [180, 181], representing an important gap in the field. One study looked at diabetic patients who subsequently developed presymptomatic dementia and reported methylation marker changes overlapping with those observed in AD [182]. Another study reported decreased peripheral telomere length in dementia patients with diabetes compared to those without [183].

### 3.8 Glymphatic Dysfunction

The glymphatic system clears neurotoxic proteins including amyloid and tau from the brain's interstitial space, and its impairment has been implicated in neurodegeneration [184, 185, 186]. In diabetic animal models, impaired tracer clearance and altered AQP4 polarity indicate disrupted glymphatic function [187, 188]. Peripheral mechanisms, including dysregulation of microRNAs, may also contribute to glymphatic disruption in diabetes [189]. Human studies have corroborated this, reporting decreased ALPS index, an indirect DTI‐based marker of glymphatic activity, in individuals with diabetes [185, 190, 191, 192].

## 4 Emerging Therapeutic Strategies in Diabetes‐Associated Cognitive Impairment

### 4.1 Therapies Targeting Metabolic and Insulin Signaling Pathways

#### 4.1.1 GLP‐1 Receptor Agonists

Glucagon‐like peptide‐1 (GLP‐1) is a pleiotropic incretin hormone that enhances insulin secretion, delays gastric emptying, and reduces appetite and food intake [193, 194]. GLP‐1 receptor RNA is expressed in several brain regions, most prominently the hypothalamus, with lower levels in the brainstem and cortex [195, 196], supporting a potential role for GLP‐1 signaling within specific brain circuits beyond its systemic effects on insulin sensitivity [193]. In animal models of AD, GLP‐1 receptor agonists (GLP‐1 RAs) showed neuroprotective benefits, such as reduced amyloid and tau pathology, attenuation of central insulin resistance [197, 198, 199, 200].

A recent large meta‐analysis of 23 clinical trials of cardioprotective glucose‐lowering agents reported a statistically significant reduction in dementia risk among individuals receiving GLP‐1RAs, but not sodium–glucose cotransporter 2 inhibitors (SGLT2is) [201]. Whether this association is independent of glycemic control remains unclear. Notably, a recent Phase 3 trial of oral GLP‐1 RA in early AD failed to show slowing of clinical progression [202].

#### 4.1.2 SGLT‐2 Inhibitors

Sodium–glucose cotransporter 2 (SGLT2) is expressed primarily in the proximal convoluted tubules of the kidney, where it mediates renal glucose reabsorption [203, 204, 205]. SGLT2is have demonstrated robust cardiorenal benefits, including favorable remodeling effects in chronic kidney disease and heart failure with reduced ejection fraction, and have been approved for these indications [206, 207]. Clinical evidence regarding cognitive outcomes with SGLT2is remains mixed (Table 1); retrospective cohort studies have reported lower dementia incidence with SGLT2i use [208, 212, 213, 214, 215, 216, 217], though this was not replicated in the meta‐analysis of randomized trials [201], and prospective RCTs are therefore needed.

**TABLE 1:** Emerging therapeutic strategies relevant to diabetes‐associated cognitive impairment.

Intervention	Study type	Cognitive & clinical outcomes or comments
*Metabolic & insulin signaling*		
GLP‐1 RA	Meta‐analysis of RCTs	Decreased dementia or cognitive impairment risk (OR 0.55; 95% CI, 0.35–0.86) [201]
	Retrospective cohort studies	Lower incidence of dementia [208, 209, 210]
	Two phase 3 RCTs (AD, oral semaglutide vs. placebo)	No significant difference vs. placebo in CDR‐SB change from baseline to week 104 (evoke *n* = 1855; evoke+ *n* = 1953) [202]
	Phase 2b RCT (AD, daily liraglutide vs. placebo injection)	No changes in cerebral glucose metabolism (primary outcome) observed, modest improvement in executive function as a secondary outcome (unadjusted analysis, no change in CDR‐SB) [211]
SGLT2i	Meta‐analysis of RCTs	No significant change in dementia risk [201]
	Retrospective cohort studies	Lower incidence of dementia [208, 212, 213, 214, 215, 216, 217]
Intranasal Insulin	Meta‐analysis of RCTs (AD/MCI)	No significant improvement in cognitive outcomes [218]
	Phase 2 RCT (DM and HC; 40 unit INI vs. placebo once daily for 24 weeks, additional 24 more weeks observation period)	No cognitive improvement in DM, though improved cognitive score in HC [219] T2D participants showed improved gait speed, increased medial prefrontal cortex cerebral blood flow, and reduced HOMA‐IR, but no significant cognitive benefit (*n* = 223)
	Phase 2 RCT (Metabolic syndrome plus MCI)	Ongoing clinical trial NCT06072963
*Vascular risk factor management and neurovascular dysfunction*		
Blood pressure management	RCTs; longitudinal cohorts	Slows progression of WMH and stroke risk; possible cognitive benefit [220, 221]
Antiplatelet therapy	RCTs	Reduces recurrent stroke risk; no evidence for primary cognitive benefit in diabetes patients in the absence of cardiovascular disease [222, 223]
Statin	Meta‐analysis of cohort and case–control studies	Modest decrease in AD and non‐AD dementia risk reported [224]; however, current indications remain primary or secondary cardiovascular prevention rather than cognitive benefit specifically [225]
Symptomatic management for Orthostatic Hypotension		Cognitive outcomes not established
*Neuroinflammation and oxidative stress*		
AGE‐RAGE	Phase 3 RCT (Azeliragon in AD)	No difference in ADAS‐Cog11, CDR‐SB, or brain atrophy at 18 months [226, 227]
Intranasal anti‐CD3	Phase 2 RCT (non‐active SPMS)	Ongoing trial NCT06292923, in expanded access program (*n* = 10) decreased microglial activation at 6 months, and stable EDSS scores at 12 months [228]
	Phase 2 RCT (mild AD)	Ongoing trial NCT06489548 [229].
*Gut‐brain axis and dietary and lifestyle modifications*		
Probiotics	RCTs (small size, AD) Meta‐analysis of RCTs	Mixed cognitive effects [230, 231] Increased BDNF levels in patients with neurological disorders [232]
Intermittent fasting	Small size RCTs (high BMI)	Modest cognitive improvements reported [233, 234]; however, no large‐scale studies have been conducted and heterogeneity in fasting regimens and outcomes limits generalizability.
Ketogenic diet	Meta‐analysis of RCTs (AD)	Improved MMSE/ADAS‐Cog; worsened lipid profile [235]
Weight loss	Meta‐analysis	Each 1% reduction in body weight increases the probability of complete remission of Type 2 diabetes by 2.17 percentage points and partial remission by 2.74 percentage points [236]

Abbreviations: ADAS‐Cog, Alzheimer's Disease Assessment Scale–Cognitive Subscale; CDR‐SB, Clinical Dementia Rating ‐ Sum of Boxes; EDSS, Expanded Disability Status Scale; GLP‐1 RA, glucagon‐like peptide‐1 receptor agonist; HC, healthy controls; MCI, mild cognitive impairment; RCT, randomized controlled trial; SGLT2i, sodium–glucose cotransporter‐2 inhibitor; SPMS, secondary progressive multiple sclerosis; WMH, white matter hyperintensity.

#### 4.1.3 Intranasal Insulin

Intranasal insulin (INI) efficiently reaches the brain [237] and modulates regional CBF (increases CBF in the inferior frontal gyrus, dorsal striatum, and insula, while reducing CBF in the middle frontal gyrus and hypothalamus), though some of these CBF effects are blunted in overweight and obese individuals [238]. Cognitive outcomes of INI are mixed (Table 1) and substantial heterogeneity was observed among the studies [239, 240], and a recent meta‐analysis of RCTs of MCI and AD patients (study range 3 weeks to 4 months) didn't identify improvement in cognitive or functional outcomes [218]. In a Phase II RCT including healthy controls and T2D patients (*n* = 223), INI improved gait speed and medio‐prefrontal cortex CBF but did not improve cognitive outcomes in diabetic patients [219].

### 4.2 Therapies Targeting Neurovascular and Endothelial Dysfunction

In symptomatic atherosclerotic disease, antiplatelet therapy together with vascular risk factor optimization represents the standard of care. However, aspirin has not demonstrated a clear cognitive benefit in diabetic patients in the absence of established cardiovascular disease [222].

In cerebral small vessel disease, particularly in patients with WMH, the benefit of antithrombotic therapy for primary stroke prevention or cognitive improvement has not been established [223, 241, 242]. Therefore, primary prevention strategies focusing on comprehensive vascular risk factor modification remain essential [223].

### 4.3 Therapies Targeting Neuroinflammation and Oxidative Stress

#### 4.3.1 AGE‐RAGE Pathway Inhibitors

In preclinical studies, RAGE antagonists FPS‐ZM1 and Azeliragon reduced amyloid pathology and neuroinflammation [243, 244]. However, azeliragon failed to demonstrate cognitive benefit in a Phase 3 trial in mild AD [226, 227].

#### 4.3.2 Modulation of Microglial Activation

If the role of microglial activation in diabetes is further established, microglia‐targeted therapies may become relevant [245]. Nasal anti‐CD3 is a promising approach; in an AD mouse model it reduced microglial activation and improved cognition independent of amyloid deposition, promoting a shift toward a homeostatic phenotype [246]. In an experimental autoimmune encephalomyelitis mouse model, it reduced both microglial and astrocytic activation through IL‐10‐producing regulatory T cells [247]. In humans, nasal anti‐CD3 has been developed as Foralumab, currently under investigation in Phase 2a trials for mild AD and non‐active secondary progressive multiple sclerosis (Table 1), with preliminary evidence of reduced microglial activation on TSPO‐PET in the latter [228].

### 4.4 Gut–Brain Axis and Microbiota‐Based Therapies

#### 4.4.1 Probiotics

The gut–brain axis is increasingly recognized as a modulator of neuroinflammation, metabolic regulation, and cognitive health. In diabetes, gut dysbiosis is characterized by altered microbial structure and changed metabolite synthesis [248, 249]. There is substantial interstudy variation; however, decreased production of short‐chain fatty acids (SCFAs) has been associated with diabetes [249, 250, 251]. SCFAs maintain BBB integrity and modulate some neurotrophic factors in experimental models [252, 253, 254]. Reduced SCFA levels have been reported in small diabetic cognitive impairment and MCI cohorts [255, 256, 257]. Probiotic interventions may mitigate DACI risk by improving glycemic control, insulin resistance, oxidative stress [232, 258, 259, 260], though clinical evidence for cognitive outcomes remains limited in AD and dedicated trials in DACI are lacking [230, 231].

#### 4.4.2 Dietary Interventions

Dietary modification is a cornerstone of diabetes management and may also influence cognitive outcomes. In animal models, high‐fat diets promote insulin resistance (i.e., PI3K/Akt overactivation), neuroinflammation, microglial activation (i.e., TLR signaling), and tau phosphorylation [261, 262, 263]. In humans, high saturated fatty acid intake has been associated with cognitive impairment [264, 265, 266], and caloric excess leading to obesity is linked to altered brain insulin sensitivity [238]. Conversely, caloric restriction and intermittent fasting (IF) increase ketone body availability and, in preclinical models, reduce oxidative stress, upregulate BDNF, and favorably modulate gut microbiota [267, 268, 269, 270]; though human data remain limited to small studies with variable results [233, 234, 271]. The opposing effects of caloric excess and high‐fat diets versus caloric restriction and intermittent fasting on shared metabolic and neuroinflammatory pathways suggest that diet may modulate risk for both DACI and AD.

Several dietary patterns, including Mediterranean, DASH, low‐fat, IF, and carbohydrate‐restricted diets, have been shown to improve metabolic parameters in diabetes; however, current evidence is insufficient to recommend one specific dietary pattern over another for optimal long‐term glycemic control or cognitive benefit [272, 273, 274, 275, 276]. Ketogenic diets, on the other hand, although modest cognitive benefits have been reported in AD, are typically high in fat and may raise LDL cholesterol and triglyceride levels, raising concerns regarding long‐term cardiometabolic safety in individuals with diabetes [235].

### 4.5 Lifestyle Interventions

Lifestyle modification plays an important role in reducing dementia risk and is particularly relevant in DACI, given that a substantial proportion of dementia cases is attributable to modifiable risk factors closely linked to diabetes, including physical inactivity, obesity, hypertension, and metabolic dysfunction [9]. Physical activity and weight reduction are especially relevant; substantial weight loss may meaningfully alter metabolic disease trajectories (Table 1) [236], and regular physical activity improves cardiovascular function, blood pressure, slow‐wave sleep, and may support brain clearance mechanisms, thereby mitigating neurodegenerative processes [185].

### 4.6 Other Potential Mechanisms

Several additional pathways have been proposed in the pathogenesis and treatment of diabetic cognitive impairment, including ferroptosis inhibition [171], modulation of autophagy [277], mitophagy and mitochondrial protection (e.g., via the humanin peptide) [278], stabilization of HIF‐1α [279], and modulation of microRNAs (miRNAs) [280, 281]. Each of these mechanisms offers promising therapeutic potential but warrants further preclinical and clinical exploration.

## 5 Discussion

DACI represents an increasingly prevalent and clinically significant complication of diabetes, highlighting the need for early clinical recognition and cardiometabolic risk factor management. Mechanistically, DACI can be conceptualized as a neurovascular‐metabolic disorder characterized by the disruption of the neurovascular unit, brain glucose metabolism, insulin signaling abnormalities, and chronic neuroinflammation. These processes lead to small vessel disease and regional brain atrophy as well as functional network dysfunction (e.g., default mode network), ultimately manifesting as cognitive impairment across a spectrum from vascular cognitive impairment to AD.

We propose that DACI represents a spectrum in which diabetes may act either as a risk factor for VaD or AD, or as a primary metabolic and oxidative stress related driver of neurodegeneration in patients where significant amyloid pathology or small vessel disease is not apparent. This concept is supported by neuropathological and PET evidence that has failed to demonstrate increased amyloid burden in individuals with diabetes [104, 118, 119, 120, 121, 122, 123, 124, 125, 126, 127, 128, 129]. We acknowledge that neurovascular dysfunction remains a central component of DACI pathophysiology and that the clinical presentation closely resembles a vascular phenotype; dedicated biomarker studies are therefore needed to distinguish cases where diabetes acts as a primary driver from those where it primarily contributes as a vascular risk factor.

Advancing the field will require biomarker strategies that reflect diabetes‐specific brain injury and help establish DACI as a distinct pathophysiological entity rather than simply a dementia risk factor. A key unanswered question is whether diabetes promotes an early form of tauopathy in a subset of patients, given that emerging CSF p‐tau and tau PET data suggest possible tau dysregulation. As with other neurodegenerative diseases, where copathology across proteinopathies is the norm [282], DACI may represent a metabolic disorder with overlapping features with VaD and AD. Toward differentiating DACI from these entities, whether cerebral glucose hypometabolism patterns can be systematically clustered into vascular, AD‐like, and diffuse metabolic phenotypes remains an important unanswered question. Longitudinal biomarker studies should specifically focus on DACI cases lacking apparent small vessel disease or amyloid pathology to characterize their long‐term trajectory. Cellular and neuroinflammatory markers, including brain‐derived extracellular vesicles, CSF transcriptomic analyses, and microglial activation assessed by TSPO‐PET, represent important and largely unexplored avenues that could further help differentiate DACI from AD or VaD.

As glucose‐lowering therapies with pleiotropic effects, particularly GLP‐1 receptor agonists and SGLT2 inhibitors, are increasingly incorporated into routine care, prospective trials specifically powered to assess cognitive outcomes in diabetes are needed. Observational studies suggesting reduced dementia risk are hypothesis‐generating and do not establish causality. Given that DACI‐related neurodegeneration is driven by neurovascular‐metabolic dysfunction, it is tempting to suggest that the cognitive benefits of these agents may be most apparent when initiated early, before irreversible neurodegeneration has occurred. This highlights the importance of optimizing diabetes management through dietary and pharmacological interventions at the earliest signs of cognitive impairment.

## Author Contributions

O.C. conceptualized the review, conducted the literature search, drafted the manuscript, and prepared the figures and tables. P.M. and H.I.C. contributed to literature review and manuscript revision. R.J. contributed to manuscript revision and supervised the project. A.I.L. supervised the project. All authors read and approved the final manuscript.

## Funding

The authors have nothing to report.

## Conflicts of Interest

The authors declare no conflicts of interest.

## Data Availability

This is a narrative review article. No new data were generated or analyzed in this study. All data discussed are derived from previously published studies cited in this manuscript.
